# The use of the mHealth program Smarter Pregnancy in preconception care: rationale, study design and data collection of a randomized controlled trial

**DOI:** 10.1186/s12884-017-1228-5

**Published:** 2017-01-26

**Authors:** Matthijs R. van Dijk, Elsje C. Oostingh, Maria P. H. Koster, Sten P. Willemsen, Joop S. E. Laven, Régine P. M. Steegers-Theunissen

**Affiliations:** 1 Department of Obstetrics and Gynecology, Erasmus MC, University Medical Centre Rotterdam, PO Box 2040, Rotterdam, 3000CA The Netherlands; 2 Department of Biostatistics, Erasmus MC, University Medical Centre Rotterdam, PO Box 2040, Rotterdam, 3000CA The Netherlands; 3 Department of Pediatrics, Division of Neonatology, Erasmus MC, University Medical Centre Rotterdam, PO Box 2040, Rotterdam, 3000CA The Netherlands

**Keywords:** Nutrition, Lifestyle, Fertility, Reproductive outcome, Pregnancy outcome, Pregnancy complications

## Abstract

**Background:**

Unhealthy nutrition and lifestyle contribute to the worldwide rising prevalence of non-communicable diseases. This also accounts for the reproductive population, in which unhealthy behavior affects fertility and pregnancy outcome. Maternal smoking, alcohol consumption and inadequate folic acid supplement use are strongly associated with fetal complications as small for gestational age, premature birth and congenital malformations. In the Netherlands 83% of the perinatal mortality rate is due to these complications and is relatively high compared to other European countries. In order to reduce this prevalence rate, preconception care should be focused on the promotion of health of prospective parents by identification and intervention on modifiable nutrition and lifestyle risk factors. We developed the personal mHealth program ‘Smarter Pregnancy’ (Dutch version available on: https://www.slimmerzwanger.nl) to provide individual coaching and information to improve nutrition and lifestyle during the preconception period in order to improve health of the reproductive population and subsequent generations.

**Methods:**

Women between 18 and 45 years of age, and trying to conceive are eligible for inclusion in a randomized controlled trial. Participants are allocated either to a general population cohort or a subfertile (IVF/ICSI) population cohort. The intervention group receives personal online coaching based on the identified nutrition and lifestyle risk factors at baseline. Coaching comprises recipes, incentives, additional questions including feedback and text and e-mail messages, with a maximum of three per week. The control group only receives one recipe per week to maintain adherence to the program and prevent drop out. Screening questionnaires are send in both groups at 6, 12, 18, and 24 weeks of the program to monitor the change in the identified risk factors.

**Discussion:**

We expect to demonstrate that the mHealth program ‘Smarter Pregnancy’ can effectively improve nutrition and lifestyle in couples contemplating pregnancy. By the identification and improvement of modifiable nutrition and lifestyle risk factors on a large scale, both reproductive and pregnancy outcomes can be improved and subsequent perinatal morbidity and mortality rates are expected to be reduced. The current use and rapid development of mHealth applications offers new opportunities to reach and educate large populations, which can facilitate the implementation of preconception care.

**Trial registration:**

Dutch trial register: NTR4150. (Registered 19^th^ August 2013)

**Electronic supplementary material:**

The online version of this article (doi:10.1186/s12884-017-1228-5) contains supplementary material, which is available to authorized users.

## Background

Unhealthy nutrition and lifestyle, characterized by a high caloric intake and vitamin deficiencies, derange metabolic and endocrine pathways and are causing obesity which contributes to the development of non-communicable diseases (NCDs), such as cardiovascular and metabolic diseases [1, 2]. Although awareness of the impact of unhealthy nutrition and lifestyle is increasing, its prevalence remains very high, not only in general, but also in the reproductive population in which health consequences range from subfertility to congenital malformations or even perinatal death [3–7]. Most evidence is available on the detrimental impact of maternal smoking, alcohol consumption and inadequate folic acid supplement use, which are strongly associated with embryonic growth and small for gestational age (SGA) and congenital malformations [8–12]. Currently, several studies that focused on the adherence of maternal dietary patterns have shown the benefits of healthy foods such as fruits and vegetables on perinatal outcome [13, 14].

In the Netherlands, particularly in large cities such as Rotterdam, perinatal mortality rates and the prevalence of perinatal complications, such as SGA, premature birth and congenital malformations (also referred to as Big3 complications), is relatively high compared to other European countries [15–17]. In order to reduce these prevalence rates, preconception care (PCC) should be implemented, focused on the promotion of health and the identification of (modifiable) risk factors of prospective parents as well as the next generation [18–20].

In order to create awareness and to implement PCC on a large scale, new approaches need to be explored and (mobile) technologies can be used. Previously, we developed and implemented a preconception outpatient clinic tailored to improve nutrition and lifestyle of which the results were promising, i.e. 30% reduction of inadequate nutrition and lifestyle and a 65% increased chance of ongoing pregnancy after IVF treatment [4, 21]. However, this outpatient clinic could only provide PCC on a small scale due to the required expertise, time and costs. To overcome these barriers we have developed the personal mHealth coaching program ‘Smarter Pregnancy’ (Dutch version available on: www.slimmerzwanger.nl, English equivalent available on: https://www.smarterpregnancy.co.uk/research/), providing individual, tailored and continuous information on a large scale during 26 weeks. Previous studies have shown that women seek online information with regard to healthy nutrition and lifestyle which suggests that online interventions using mobile technology can be effective [22, 23]. Also, women embrace online anonymity to control and self-manage online information [24, 25]. Smarter Pregnancy identifies the most important risk factors regarding nutrition and lifestyle and subsequently provides tailored information and motivational coaching by text and e-mail messages [6].

We hypothesize that our mHealth program will effectively improve nutrition and lifestyle in couples contemplating pregnancy. Based on our previous studies and that of others we designed a randomized controlled trial (RCT) to study the effectiveness of “Smarter Pregnancy”, defined as a significant improvement of vegetable and fruit intake and folic acid supplement use, when started preconceptional [4, 6, 7, 21, 26]. This intervention can be considered as a primary prevention tool resulting in a reduction of Big-3 complications, perinatal morbidity and mortality in the short-term and NCDs in the long-term [2, 27, 28].

### Objectives

A randomized controlled trial is conducted in two independent populations, i.e. couples from the general population and couples undergoing IVF/ICSI treatment, to study whether unhealthy nutrition and lifestyle can be improved by the Smarter pregnancy coaching program as an intervention tool. Furthermore, we will determine whether couples will have a higher pregnancy rate and if their risk for Big3 complications can be reduced by improving nutrition and lifestyle.

#### Primary outcome

Improvement (percentage reduction) of unhealthy nutrition and lifestyle in women and men contemplating pregnancy or already pregnant, determined by using a dietary risk score (DRS), 24 weeks after starting the “Smarter Pregnancy” intervention.

#### Secondary outcomes

1) A reduction in smoking by women and men contemplating pregnancy; 2) pregnancy rates in couples; 3) birth prevalence rate of Big-3 complications in the entire study population; 4) cost-effectiveness of the Smarter Pregnancy intervention.

#### Tertiary outcomes

The influence of participation of men, pregnancy, age, low socioeconomic status on the primary outcome and 1) Improvement (defined as the percentage of reduction) of unhealthy nutrition and lifestyle 36 weeks after starting the “Smarter Pregnancy” intervention; 2) the compliance and reliability of “Smarter Pregnancy” among both women and men. To study the latter, we aim to determine the: 1) The percentage of the target group that meets all the inclusion criteria for the study, but does not participate; 2) The percentage of participants that is still participating after three months (compliance); 3) The prevalence and nature of technical problems.

## Design

### Eligibility

Women residing in the Netherlands who are between 18 and 45 years of age and contemplating pregnancy are considered eligible to be included in this study. To participate, women need to be in possession of a smartphone with Internet access. Women with insufficient knowledge or understanding of the Dutch language, women who are treated by a dietician to lose weight in the context of a fertility treatment, and women who have a specific diet (e.g. vegans) cannot participate in the study. Male partners are also invited to participate, but only if they meet the same criteria, except that there is no upper age limit for male participants.

### Recruitment, cohort composition and randomization

Women are invited to participate by a (health care) professional from their midwifery practice, children’s daycare, childhealth center, or hospital. Self-registration through the website is also possible. Potentially eligible participants are contacted after registration by one of the researchers to verify their eligibility, to provide more details and answer questions about “Smarter Pregnancy” and to confirm their registration.

Participants are allocated either in a general population cohort or the IVF/ICSI- (ART) population cohort, depending on whether they will receive fertility treatment. Randomization of the participants is stratified by cohort and per center of inclusion. For each stratum a permuted block design is used and programmed beforehand. Hereby, allocation concealment is ensured.

### Smarter pregnancy

The mHealth program Smarter Pregnancy was launched in 2012 and provides personal coaching, tailored on personal conditions, gender, nutrition and lifestyle in both women and men contemplating pregnancy. The program is based on nearly 30 years of research and expertise by our group on the influence of nutrition and lifestyle on reproduction and pregnancy course and outcome. We used elements of Prochaska and Diclemente’s transtheoretical model with a focus on the readiness for behavioral change, Bandura’s social cognitive theory for self-efficacy and Fogg’s behavior model to include triggers to motivate and increase the ability to change [29–31]. Features of the attitude, social influence, and self-efficacy (ASE) model for coaching are applied; aimed at the understanding and motives of people to engage in specific behavior [32].

### Intervention group

The content of the individual coaching is based on the baseline screening on personal conditions, nutrition and lifestyle and monitoring questionnaires at 6, 12, 18, and 24 weeks of the Smarter Pregnancy program. At these time points, participants are invited to complete a short, online questionnaire to monitor the change in their nutrition and lifestyle. Results from the questionnaires are compared with the previous results and shown on a personal online page to show a participant’s progress.

**Fig. 1:**
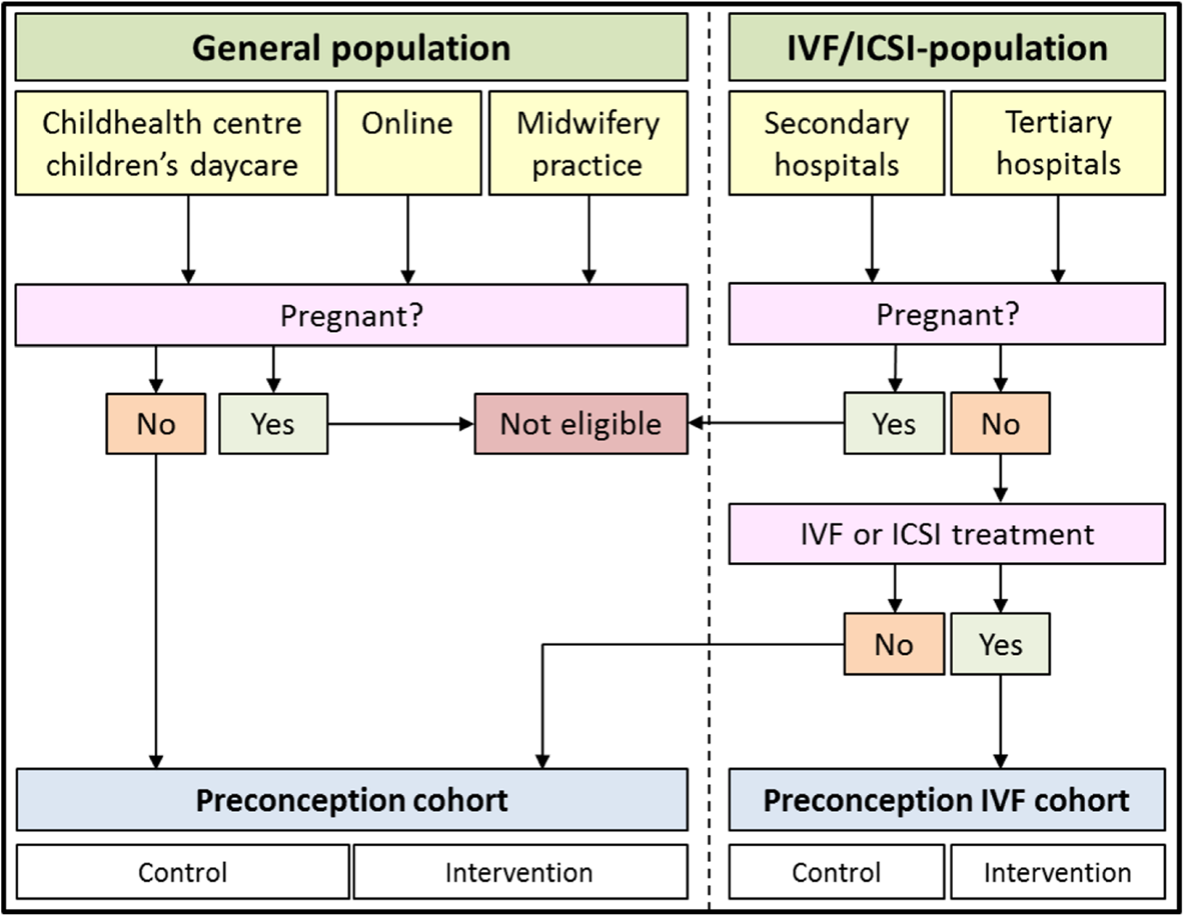
Overview of the recruitment and composition of the multi-center study and both cohorts

The tailored coaching includes a maximum of three interventions per week comprising short message service (SMS) text and email messages containing tips, recommendations, vouchers, seasonal recipes, and additional questions addressing behavior, pregnancy status, body mass index (BMI) or adequacy of the diet (Fig. 1, colored arrows above black arrows).

The personal page also provides access to additional modules (i.e. applications) to support physical activity, an agenda to improve the compliance with hospital appointments and medicine adherence, and a module to monitor the safety of prescribed medication. A summary of all individual results can be obtained at any moment by the participant, and can be handed over or sent by email to the health care professional for further evaluation and support of preconception and antenatal care.

### Control group

Participants who are randomized in the control group will not receive personal coaching after the baseline screening. They do receive access to their personal page and will receive one seasonal recipe per week to maintain adherence and prevent drop out (Fig. 1, lower red arrows). At baseline as well as at 12 and 24 weeks, participants in the control group receive the monitoring questionnaire about nutrition and lifestyle, but without feedback on the results. Also, every 6 weeks the controls receive a request to adjust their pregnancy status if needed.

### Biomarker validation

To validate the self-administered questionnaires, we will analyze several blood biomarkers in a random sample of both study populations and both groups (intervention and control group). A team of qualified medical students will take blood samples at the participants home address or at the hospital. These blood samples will be taken on three time points (t = 0, 12 and 24 weeks) during the study; each time 20 ml will be collected. Samples are kept at −20° Celsius for a maximum time period of 4 h. Aliquots of residual blood will be stored at −80° Celsius for future research on DNA and epigenetics.

### Additional study questionnaires and follow-up

**Fig. 2:**
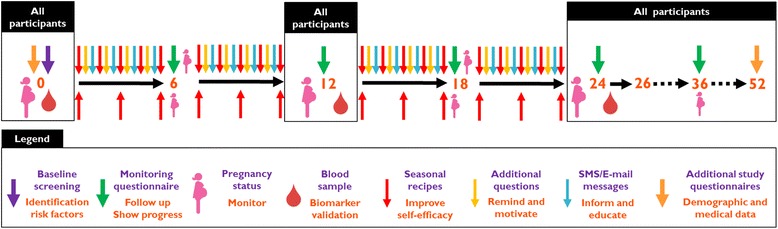
Overview of both the intervention and control group during their enrollment. The upper arrows pointing downwards depict the intervention group. The lower arrows pointing upwards depict the control group. All boxed icons depict aspects of the trial that account for all participants in both groups, i.e. baseline screening, screening questionnaires (at t = 12 and t = 24 weeks), additional questionnaires at baseline and 52 weeks, pregnancy status per 6 weeks and blood samples (see Additional file 1: SPIRIT-Table)

At baseline, for both the intervention and the control group additional information on social and demographic characteristics is obtained using an additional online study questionnaire implemented in the coaching program. The first follow-up study questionnaire will be send at 36 weeks, i.e. 12 weeks after the last screening moment (Fig. 2). One year after enrollment, participants receive their last study questionnaire, which consists of questions regarding medical and obstetric history, medication use, whether they became pregnant during enrollment and, if applicable, the pregnancy outcome.

### Statistical considerations

Sample size calculations are based on our primary outcome measure (DRS). Based on our previous studies and the survey using Smarter Pregnancy, we expect a reduction of approximately 0.5 DRS points (based on a standard deviation of 2.7) in the intervention group compared to the control group. Considering alpha = 0.05 and power = 0.80 we will need to include a total of 916 women in our study (2 arms of 458 each). Due to expected drop outs of approximately 10%, we aim to include 1000 fertile (2 arms of 500 each) and 1000 subfertile women (2 arms of 500 each) in our study. For 50% of these women, we expect their male partner (*n* = 250 in each arm) to participate as well. Due to the lower SD (2.0) in men, with this sample size we are also able to demonstrate a reduction of at least a 0.5 DRS points in the male partners.

### Statistical analysis

A flowchart will be used to depict the total participants of each cohort and divided per group, subdivided per gender. Also, the amount of resigning participants will be shown per time point (6 weeks). General and baseline characteristics will be compared between groups and shown in a baseline table.

The primary analysis will be based on intention to treat (ITT). For men and women in both the intervention and the control group the DRS will be calculated at baseline and after 24 weeks and used for further analyses. This continuous outcome measure will be analyzed by the ‘difference in difference principle’ and used in a linear regression model, including the initial/baseline value of the DRS. Repeated measurements will be used to investigate the effects of the intervention over time and the interaction of the intervention with socio economic status, ethnicity and age. Chi-square analysis and ANCOVA will be used to study the effects of the intervention on the pregnancy outcome and Big-3 complications.

To measure the compliance and reliability of ‘Smarter pregnancy’ we will analyze the percentage of randomized women who fill in the questionnaire after 12 weeks of participation and the percentage of participants who experienced technical problems. Corresponding confidence intervals will be given.

The influence on the primary outcome of participation of men, if pregnancy occurred during participation, age and low socio economic status will be analyzed by including these variables and their interaction with both groups, one by one in the model which will be used for the primary outcome. If there is heterogeneity of the treatment effect, the effect will be determined per subgroup separately.

## Discussion

This study will contribute to the implementation of easily accessible PCC in order to increase awareness regarding the importance of healthy nutrition and lifestyle in couples contemplating pregnancy and health care professionals. Subsequently, this can reduce the relatively high rates of perinatal morbidity and mortality (Big3 complications) in the Netherlands.

Initiating behavioral change(s) by the identification of risk factors during the preconception period can be a useful first step to not only create awareness, but also to lower the threshold to approach a healthcare professional during this period. Discussing or revealing involuntary childlessness remains a burden for many women as well as for men, due to the perception that they have failed by not being able to conceive. This results in a situation in which risk factors for poor reproductive and pregnancy outcome persist, while adopting a healthy lifestyle during this preconception period can be beneficial on both the short and long term. Most reproductive failures originate due to deranged metabolic pathways. The lack of co-factors and substrates as a result of vitamin deficiencies (e.g. vitamin B12 and folate) can influence oocyte en semen quality and early embryonic development resulting in failed implantation and miscarriages. Also, it can cause epigenetic modifications to DNA methylation of the offspring [19, 33, 34]. Therefore, we consider the preconception period as the window of opportunity to initiate a healthy lifestyle.

Currently, research in the field of mobile technology is mainly aimed on the use of mHealth in low- and middle-income countries, because this new form of health care delivery can reach the poorest regions in which the prevalence of NCDs and poor maternal and child health are the highest [35–38]. By our opinion, also high income countries comprise specific target groups, such as the reproductive population, in which risks for poor reproductive and pregnancy outcome accumulate, because of the lack of knowledge and self-efficacy with regard to PCC [39]. Therefore, we consider mHealth a promising method to approach the large group of reproductive women and men which is currently wrongly assumed to be at low risk for poor reproductive and pregnancy outcome, although it is known that the prevalence of risk factors in this population is high [4, 6, 11]. Given that 98.6% of all Dutch women and men between 18 and 45 years old have access to the internet and 96.2% can access the internet by their mobile phone makes this mHealth approach justifiable [40].

Strengths of this RCT are the longitudinal observations and the longitudinal biomarker validation in blood. Also, additional study questionnaires for short-term and long-term follow-up (respectively 12 and 26 weeks after the last questionnaire at 24 weeks), including sociodemographic data and medical record validation, are considered important strengths of this study. A limitation of this RCT is the potential selection bias, which is unfortunately inherent to participation in a study, especially on behavioral change, as well as the exclusion of participants without sufficient knowledge of the Dutch language.

With this RCT we expect to demonstrate the effectiveness of our Smarter Pregnancy program and its positive effect on reproductive and pregnancy outcome in both fertile and subfertile couples. Healthcare professionals are often also not aware of the importance of PCC nor have tools containing information and guidelines to provide nutrition and lifestyle care for medical practice [41]. Therefore, we consider this study a unique intervention regarding the implementation of accessible preconception care.

## Additional file

Additional file 1: SPIRIT-Table. (DOCX 20 kb)

## Abbreviations

ART: Assisted reproductive therapy
ASE-model: Attitude social influence and self-efficacy model
BMI: Body mass index
CE-1: Conformité Européenne classe 1
DNA: Deoxyribonucleic acid
DRS: Dietary risk score
ICSI: Intracytoplasmic sperm injection
ITT: Intention to treat
IVF: In vitro fertilization
mHealth: Mobile health
NCD: Non-communicable disease
PCC: Preconception care
PIF: Patient information form
RCT: Randomized controlled trial
SD: Standard deviation
SGA: Small for gestational age
SMS: Short message service
