# Supplementary material for: The use of the mHealth program Smarter Pregnancy in preconception care: rationale, study design and data collection of a randomized controlled trial
Source: BMC Pregnancy Childbirth. 2017 Jan 26;17:46. doi: 10.1186/s12884-017-1228-5 (PMC5270226; doi:10.1186/s12884-017-1228-5)
Supplement: Additional file 1: — SPIRIT-Table. (DOCX 20 kb) [file 12884_2017_1228_MOESM1_ESM.docx]

**SPIRIT-Table**

|  | **Study period** | | | | | | | |
| --- | --- | --- | --- | --- | --- | --- | --- | --- |
|  | **Enrollment** | **Allocation** | **Post-allocation** | | | | | **Close-out** |
| **Timepoint (weeks)** |  | t_0_ | t_6_ | t_12_ | t_18_ | t_24_ | t_36_ | t_52_ |
| Eligibility screen | X |  |  |  |  |  |  |  |
| Informed consent | X |  |  |  |  |  |  |  |
| Allocation |  | X |  |  |  |  |  |  |
| **INTERVENTION GROUP** |  |  |  |  |  |  |  |  |
| *Screening questionnaire* |  | X | X | X | X | X | X |  |
| *Additional questionnaire* |  | X |  |  |  |  |  | X |
| *Coaching* |  |  |  |  |  |  |  |  |
| *Pregnancy status* |  | X | X | X | X | X | X |  |
| *Blood collection** |  | X |  | X |  | X |  |  |
| **CONTROL GROUP** |  |  |  |  |  |  |  |  |
| *Screening questionnaire* |  | X |  | X |  | X | X |  |
| *Additional questionnaire* |  | X |  |  |  |  |  | X |
| *Coaching* |  |  |  |  |  |  |  |  |
| *Pregnancy status* |  | X | X | X | X | X | X |  |
| *Blood collection** |  | X |  | X |  | X |  |  |
| **ASSESSMENTS** |  |  |  |  |  |  |  |  |
| Baseline   - Age - Length - Weight - BMI - Vegetable intake - Fruit intake - Folic acid supplementation - Smoking - Alcohol consumption - Pregnancy status - Physical activity - Demographics |  | X |  |  |  |  |  |  |
| Outcome variables   - Weight - BMI - Vegetable intake - Fruit intake - Folic acid supplementation - Smoking - Alcohol consumption - Pregnancy status - Physical activity |  | X | X | X | X | X | X |  |
| Blood collection   - Nutrients |  | X |  | X |  | X |  |  |
| Follow-up   - Medical history - Pregnancy outcome |  |  |  |  |  |  |  | X |

** Determined in a random sample.*

*BMI, body mass index; RBC, red blood cell count; Hb, hematoglobin; Ht, hematocrite.*
